# Le lipome para testiculaire: à propos d'un cas rare

**DOI:** 10.11604/pamj.2015.20.311.6434

**Published:** 2015-03-31

**Authors:** Abdelouahed Lasri, Hamza Lamchahab, Mounir Lahyani, Tarik Karmouni, Khalid Elkhader, Abdellatif Koutani, Ahmed Ibn Attya Andaloussi

**Affiliations:** 1 Service d'Urologie B, CHU Ibn Sina, Rabat, Maroc

**Keywords:** Lipome, tumeur para testiculaire, tumorectomie, Lipoma, testicular lipoma, lumpectomy

## Abstract

Les tumeurs para testiculaires sont des tumeurs rares et complexes qui ont une symptomatologie insidieuse et pauvre. Les formes bénignes représentent 70%. Le lipome para testiculaire étant le type le plus fréquent. L'examen clinique est peu spécifique. L'examen échographique est la pierre angulaire pour assoir le diagnostic. Le traitement chirurgical s'impose en cas de tumeur symptomatique, l'histologie étant habituellement typique permet la confirmation diagnostique. Le pronostic est bon malgré d’éventuelles récidives.

## Introduction

Parmi les tumeurs affectant le scrotum et son contenu, les néoplasies testiculaires sont les plus fréquentes. La localisation para testiculaire ne représente que 7% des localisations néoplasiques intra scrotales, 70% d'entre elles sont bénignes. Le lipome est le type le plus fréquent. Nous rapportons un nouveau cas de lipome para testiculaire chez un patient de 42 ans ayant été confirmé par l’étude anatomopathologique d'une pièce de tumorectomie. Notre travail inclut une revue de littérature avec une mise au point sur les aspects diagnostiques et thérapeutiques.

## Patient et observation

**Figure 1:**
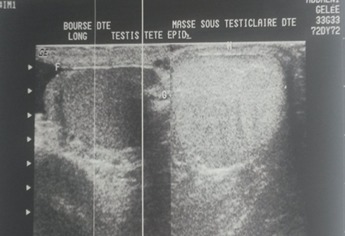
Echographie de lipome para testiculaire: masse sous testiculaire hétérogène de 4cm/3cm

**Figure 2:**
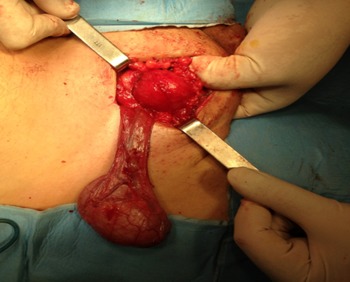
Aspect macroscopique per-opératoire montrant une masse jaunâtre, de consistance molle et disséquée du cordon spermatique

**Figure 3:**
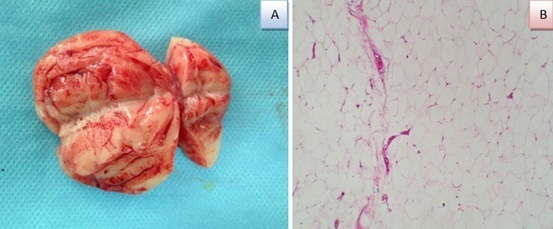
(A) aspect macroscopique: masse encapsulée avec de fines cloisons; (B) image microscopique montrant une prolifération bénigne d'architecture lobulée, faite d'adipocytes matures, à cytoplasme clair et sans atypies

Mr. B.B, âgé de 42 ans, sans antécédents, qui présentait une augmentation du volume scrotal depuis 4 ans sans notion de traumatisme ni d'atteinte inflammatoire avec une douleur modérée. L'examen physique avait retrouvé un bon état général, avec une masse scrotale droite de consistance élastique, indolore, de 5 cm, bien définie et individualisable du testicule droit qui est d'aspect normal sans nodule ni épaississement. L'hémi scrotum gauche était normal et les aires ganglionnaires étaient libres. L’échographie scrotale avait objectivé un testicule droit de taille et d’écho structure normale, refoulé par une volumineuse masse discrètement hétérogène, bien limitée et sans calcifications ni plages de nécroses mesurant 4 cm de hauteur sur 3 cm d’épaisseur. Le scrotum controlatéral était sans anomalies (Figure 1). les marqueurs tumoraux (alpha-foetoprotéine, bêta-gonadotrophine Chorionique et le lactate déshydrogénase) sont revenus normaux. Une tumorectomie par voie inguinale a été réalisée après dissection du cordon et du testicule droit de la masse qui était bien limitée, de consistance lipomateuse (Figure 2). L’étude anatomopathologique avait objectivé une masse pesant 25 gr, mesurant 5cm de diamètre, d'aspect jaunâtre et de consistance molle avec une fine capsule. A l'examen histologique, il existait une prolifération tumorale bénigne faite d'adipocytes disposées en lobules, de dimensions variables, séparées par des cloisons conjonctives (Figure 3). Apres un an de suivi, le patient est asymptomatique ne présentant pas de signes de récidive tumorale.

## Discussion

La région para testiculaire est une zone anatomique complexe qui inclut le cordon spermatique, les tuniques testiculaires, l’épididyme et les vestiges embryonnaires. Histologiquement, cette région contient une variété de tissus (épithélial, mésothélial et mésenchymateux) qui explique l'hétérogénéité des tumeurs qui peuvent s'y développer. La localisation para testiculaire ne représente que 7% des localisations néoplasiques intra scrotales. Le cordon spermatique est atteint dans 90% des cas [1, 2]. Environ 70% des tumeurs para testiculaires sont bénignes. Le Lipome est la tumeur bénigne la plus commune des tissus para testiculaires à travers tous les âges [3].

C'est une tumeur d'origine mésenchymateuse, dérivée de structures du cordon spermatique, formé par la prolifération du tissu adipeux [4]. Retrouvé à toute tranche d’âge, Leur développement est le plus souvent unilatéral, rarement bilatérale, de taille variable [5]. Cliniquement, le lipome est peu symptomatique, se manifestant par une sensation de plénitude scrotale, avec une augmentation rapidement progressive du volume scrotal, sans notion de traumatisme antérieur ni de signes inflammatoires. L'examen clinique retrouve une masse scrotale, ou inguinale de consistance molle, fluctuante et parfois multilobée. Cependant un cas de tumeur dure, de consistance pierreuse a été décrit [6]. Mais les données de l'examen clinique ne peuvent pas préjuger de la bénignité ou de la malignité des lésions et aucun signe n'est pathognomonique. Dans le doute d'une tumeur testiculaire, le dosage de marqueurs tumoraux (alpha-FP, bêta-HCG) est toujours demandé. L'examen échographique est la pierre angulaire devant tout syndrome de masse scrotale. L'aspect échographique des lipomes para testiculaires est similaire à celui observé dans le reste du corps, d’échogénéicité variable, habituellement hyperéchogène ou montrant des stries hyperéchogènes, si la masse est volumineuse ou présentant des signes de nécrose, il est difficile de la différencier d'un liposarcome [7, 8]. Dans ce cas, il parait utile le recours à d'autre moyens d'imagerie, la nature graisseuse de la masse est toujours reconnaissable: en TDM, la densité spontanée de celle-ci est inférieure à -30 Unités Hounsfield. L'IRM, montre un signal hyper intense en pondération T1, alors qu'en T2, elle présente un signal intermédiaire en écho de spin classique et un hyper signal en écho de spin rapide. En cas de doute diagnostique, une séquence avec effacement de la graisse permet le plus souvent de résoudre le problème en montrant une diminution du signal de la lésion [9].

Le traitement chirurgical s'impose en cas de tumeur symptomatique, il consiste en une tumorectomie après libération du testicule et du cordon spermatique. L'histologie est habituellement typique des lipomes trouvés dans d'autres zones du corps, avec un aspect de surface jaune-orange, lobulaire, irrégulier à la coupe. La lésion est bien délimitée et séparée des autres structures par une capsule mince. Au microscope, les cellules sont des adipocytes matures, avec un cytoplasme clair et volumineux, séparées par de fines cloisons fibreuses [10]. Ces tumeurs sont de bon pronostic et aucun cas de dégénérescence maligne n'a été rapporté. En revanche des cas de récidives ont été décrits [11].

## Conclusion

Le Lipome est la tumeur bénigne la plus commune des tissus para testiculaires, cliniquement peu symptomatique et de diagnostic tardif. La tumorectomie s'impose et l'examen anatomopathologique affirme le diagnostic. Le pronostic est bon.
